# Decorin, Tenascin C, Total Antioxidant, and Total Oxidant Level Changes in Patients with Pseudoexfoliation Syndrome

**DOI:** 10.1155/2018/7459496

**Published:** 2018-07-08

**Authors:** Yavuz Oruc, Sinem Keser, Elif Yusufoglu, Fatih Celik, İbrahim Sahin, Meltem Yardim, Suleyman Aydin

**Affiliations:** ^1^Department of Ophthalmology, Elazig Research and Education Hospital, Health Science University, 23119 Elazig, Turkey; ^2^Department of Medical Biochemistry and Clinical Biochemistry, Firat Hormones Research Group, Medical School, Firat University, 23119 Elazig, Turkey; ^3^Department of Medical Biology, Medical School, Erzincan Binali Yildirim University, 24100 Erzincan, Turkey

## Abstract

**Purpose:**

Pseudoexfoliation syndrome (PEX) is an eye disease that develops under the influence of regional population differences, genetic factors, age, and environmental factors and is characterized by visualization of a gray-white fibrogranular substance in the lens anterior capsule and/or pupil margin during anterior segment examination. The underlying biochemical mechanisms of the disease have not yet been fully elucidated. Therefore, this study was designed to show the changes in aqueous humor and blood serum levels of matrix metalloproteinases (decorin and tenascin C), total antioxidants (TAS), and total oxidants (TOS) in both cataract patients who have unilateral PEX material and cataract patients who do not have unilateral PEX material.

**Methods:**

Biological samples were simultaneously collected from 22 cataract patients who had unilateral pseudoexfoliation (PEX patients) and 22 cataract patients who did not have unilateral pseudoexfoliation (control patients). From the collected biological samples, decorin (DEC) and tenascin C (TN-C) were measured with the enzyme-linked immunosorbent assay (ELISA) method, and TAS and TOS were measured with an autoanalyzer.

**Results:**

When decorin, tenascin C, and TOS values of PEX patients were compared with those of control patients, there was a statistically significant increase in all three parameters. Conversely, TAS values showed a statistically significant decrease in PEX patients compared to controls. DEC, TN-C, TAS values, and TOS values were significantly higher in aqueous fluid than in blood in both the PEX patient and control groups.

**Conclusions:**

We suggest that parameters such as DEC, TN-C, TAS, and TOS play a role in the etiopathology of pseudoexfoliation syndrome. Thus, bringing these increased levels of extracellular proteins and TOS and decreased levels of TAS back to within physiological limits can mediate the reorganization of the blood-aqueous fluid barrier and slow the progression of pseudoexfoliation syndrome.

## 1. Introduction

Pseudoexfoliation syndrome (PEX) is a systemic disease with a 5–25% prevalence in all societies worldwide (genetically linked and influenced by race, age, and environmental factors). PEX is characterized by a grayish-white, bran-like fibrillar extracellular material in the intraocular and extraocular structures such as the pupillary flank, lens anterior capsule, iridocorneal angle, ciliary body and zonules, anterior hyaloid face, trabecular meshwork, corneal endothelium, and lid conjunctiva [1–3].

However, although the abovementioned features of PEX are known, the definitive etiopathology has not been fully elucidated. Studies to date have reported that this condition is associated with extreme accumulation of abnormally structured extracellular material in intraocular and ectodermal tissues [4]. Therefore, associations between molecules such as elastin, fibronectin, amyloid, fibrilin-1, and PEX were investigated, and their relationship was revealed. In addition, other studies have reported increased growth factors in the aqueous humor of patients with PEX. Another study group (Schlotzer-shrehud et al.) reported that the concentrations of matrix metalloproteinases and their inhibitors in the aqueous humor of PEX patients were increased [5].

Two of the most important members of the matrix metalloproteinase family are decorin (DEC) and tenascin C (TN-C). DEC is a proteoglycan that is rich in leucine amino acids. This protein is associated with collagen fibrils from all connective tissues, and the average molecular weight ranges from 90 to 140 kilodaltons (kDa). The main function of DEC is to regulate cellular proliferation and differentiation in newly formed bone [6]. DEC also mediates the stabilization of the collagen skeleton, supporting ligaments and tendons. Histochemical and immunochemical studies have reported that glycoprotein/proteoglycan molecules play a role in the etiopathology of PEX [7–14]. Thus, we believe that it is scientifically worthwhile to show whether decorin, a proteoglycan-structured molecule as described above, is involved in the etiopathogenesis of PEX. According to an extensive literature review, there is no scientific study investigating this linkage.

Similarly, TN-C is one of the glycoprotein/proteoglycan-forming matrix metalloproteinases, and a study investigating its association with PEX has not yet been performed. TN-C induces smooth muscle cell proliferation in collaboration with growth factors. The molecular weight of TN-C ranges from 250 to 300 kilodaltons (kDa). Glycoproteins and proteoglycans encircle the retinal cells. TN-C, a proteoglycan, is a key molecule in the immune system and neuroinflammatory processes. TN-C also plays a role in the etiopathogenesis of macular degeneration, diabetic retinopathy, glaucoma, and retinal vascular occlusion [15]. It has been studied in many biological fluids, cartilage extracts, synovial fluid, and blood. TN-C has not yet been studied in the aqueous humor [16, 17].

Pseudoexfoliation is a syndrome associated with cataracts. Thirty-five percent of cataract formation is caused by genetic factors, and the other 65 percent is caused by environmental factors such as nutrition, age, alcohol, cigarette consumption, diabetes, and excessive daylight exposure [18]. Antioxidants protect cells from free radicals. There is evidence that abundant intake of antioxidant vitamin C reduces the risk of cataracts, slows down cataract progression, and reduces the need for cataract surgery [19, 20]. This means that the coexistence of PEX and cataracts is one of the oxidative stress states in which antioxidant mechanisms are inhibited.

Oxidative damage has been demonstrated to play a major role in ocular diseases including cataracts and macular degeneration [21, 22]. Increasing evidence indicates that oxidative stress plays a key role in the pathogenesis of PEX syndrome [23–27]. Oxidative stress causes the production of free radicals, which are reactive chemical species generated during normal metabolic processes that can damage lipids and proteins when present in excess [28].

Therefore, it is also within the objectives of this work to establish whether total antioxidant capacity (TAS) and total oxidant capacity (TOS) have links to PEX. The diagnosis of PEX is usually made with a biomicroscope. However, this does not provide clues into the etiopathogenesis of the disease. It is important to examine aqueous humor and blood materials to reveal the biological mechanisms underlying PEX. Therefore, this study was designed in order to show whether there is a relationship between TAS or TOS and PEX and determine the changes in the levels of matrix metalloproteinases (DEC and TN-C), TAS, and TOS in aqueous humor and serum in cataract patients with unilateral PEX material and in cataract patients who do not have unilateral PEX material.

## 2. Material and Method

This study was approved by the Noninterventional Ethics Committee of Firat University on 5 January 2018 with decision number 10; informed consent forms were obtained from the subjects. The study was originally planned to be performed with 30 patients who had unilateral PEX and cataracts together (PEX group) and 30 patients with cataracts who did not have unilateral PEX (control group). However, 8 patients from each group left the study; therefore, 22 subjects for each group remained in the analysis. Patients were evaluated before surgery, and age, sex, and cataract types were recorded. The type of surgery performed (phacoemulsification, extracapsular lens extraction, intracapsular lens extraction, and insertion of the intraocular lens), approach (into the lens capsule, sulcus, anterior chamber, and fixation to sclera or aphakia), and occurrence of any complications during surgery (posterior capsule opening, zonular dialysis, drop of lens parts in the vitra, and vitreous loss) were assessed as described previously. Participants with diabetes, systemic arterial hypertension, systemic vasculopathies, retinal diseases (macular degeneration, etc.), ocular surgery, ocular trauma, or ocular inflammation were excluded from the study. In patients with PEX, the material was observed and detected on the biomicroscope at the edge of the pupil and on the anterior capsule of the lens. All of the patients included in this study were operated on using the phaco method.

A blood sample and 0.1 mL aqueous humor sample taken from the anterior chamber during cataract surgery were simultaneously collected into biochemistry centrifuge tubes and delivered to the biochemical laboratory within an ice basket. After samples were centrifuged at 4000 rpm, they were stored at −80°C. TN-C levels (Human TN-C) in the blood and aqueous humor (anterior chamber fluid) of PEX patients and control group were studied with the ELISA method (catalog number 201-12-1415 Sunred Biological Technology Co., Ltd., Shanghai, China) in accordance with the operating procedures specified in the kit instructions. The assay range for the Human TN-C ELISA kit was 200–60000 pg/mL; intraassay CV value was <10%, interassay CV value was <12%, and sensitivity was 180.556 pg/mL. As aqueous humor samples were diluted with a 1/2 ratio of pH 7.4 phosphate buffer, values for anterior chamber fluid results were entered by multiplying by a dilution factor of 2. Plate washes were performed with an automatic washer Bio-Tek ELX50 (BioTek Instruments, USA) and absorbance readings were made with ChroMate Microplate Reader P4300 instruments (Awareness Technology Instruments, USA). Test results were reported in pg/mL.

DEC levels (human decorin) in the blood and aqueous humor (anterior chamber fluid) of PEX patients and control patients were studied with the ELISA method in accordance with the operating procedures specified in the kit instructions (catalog number 201-12-3612 Sunred Biological Technology Co., Ltd., Shanghai, China). The assay range for the Human DEC ELISA kit was 0.2–40 ng/mL; intraassay CV value was <10%, interassay CV value was <12%, and sensitivity was 144 ng/mL. As aqueous humor samples were diluted with a 1/2 ratio of pH 7.4 phosphate buffer, values for anterior chamber fluid results were entered after multiplying by a dilution factor of 2. Plate washes were performed with an automatic washer Bio-Tek ELX50 (BioTek Instruments, USA) and absorbance readings were made with ChroMate Microplate Reader P4300 instruments (Awareness Technology Instruments, USA). Test results were reported in ng/mL.

TN-C measurements were initially given in pg/mL, per the manufacturing company, but were converted to ng/mL for easy comparison with DEC values. In this study, TAS and TOS values were measured according to the previously mentioned method. As TAS and TOS kits were produced in order to measure blood TAS and TOS levels, a validity test was carried out in order to see whether these kits could measure TAS and TOS levels from the aqueous humor.

TAS was measured using a fully automatic method that detects the TAS and produces a Fenton-type reaction with oxygen peroxide and Fe^+2^ o-dianisidine, resulting in OH radicals [29]. This strongly reactive oxygen species is reduced and reacts with the o-dianisidine molecule at acidic pH to form yellow-brown dianisidine radicals. These radicals produce color formations by participating in oxidation reactions. However, antioxidants present in these samples suppress these oxidation reactions and stop color formation. These reaction results are obtained automatically with an autoanalyzer measurement.

TOS is a fully automated system that oxidizes the ferrozine o-dianisidine complex to the ferric ion, showing the TOS with the uptake of the oxidants present in the material; ferric ions form a colored complex with xylenol orange at an acidic pH [30]. The intensity of the color, which is associated with the level of oxidants present in the samples, is measured spectrophotometrically. The mmol value of the TAS test was converted to *μ*mol units as in the TOS test, and the oxidative stress index (OSI) was calculated according to the following formula: OSI (arbitrary unit) = TOS (*μ*mol H_2_O_2_ Eq/L)/TAS (*μ*mol Trolox Eq/L) [31].

The intraassay CV value of TAS was <10%, while the intraassay CV value of TOS was <12%. Interassay CV values of TAS and TOS were detected as <15%. The intraassay CV value of decorin was <10% and the interassay CV value was detected as <15%. The recovery results of the kits were ranged between 98 and 114%. It was recorded that there was linearity in the measurements across dilutions. According to all the validity tests, the kits used in this study were determined to have the same sensitivity in the measurements of eye liquid as those of the blood.

### 2.1. Statistical Analysis

Differences in the preoperative distribution of age, sex, cataract type, type of operation, and complication rate during surgery were examined between groups. Statistical analyses were performed using the SPSS 22.00 package program. The Mann–Whitney *U* test was used for comparison between groups. The difference between the groups was considered significant when *p* was < 0.05.

## 3. Results

The demographic characteristics of the subjects are summarized in Table 1. There was no statistically significant difference between the PEX and control groups with regard to body mass index and age. Within the PEX group, 19 (43.2%) were male, and 25 (56.8%) were female. Within the control group, 23 (52.3%) were male and 21 (47.7%) were female. When the groups were examined according to preoperative cataract types, it was found that nuclear cataracts were seen most frequently in both groups. According to the eye symmetry, 11 (45.8%) of the cataracts were in the right eye, and 13 (542%) were in the left eye in the group without PEX (*n*=24, 54.55%). However, in PEX group (*n*=31, 70.45%), 17 of the cataracts were in the right eye (54.8%), and 14 of them were in the left eye (45.2%) according to eye symmetry. There was a significant difference in the distribution of cataract type according to the groups (*p*=0.02) (Table 1).

**Figure 1:**
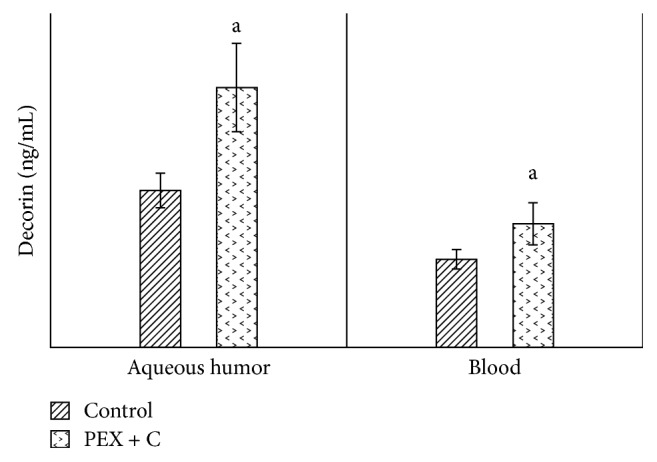
Comparison of decorin levels in intraocular fluid and blood between patients with and without pseudoexfoliation syndrome. C: cataract; PEX: unilateral pseudoexfoliation syndrome. (a) *p* < 0.05.

**Figure 2:**
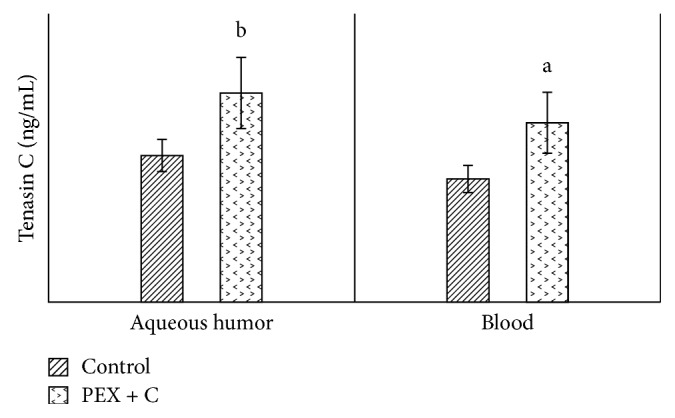
Comparison of tenascin C levels in aqueous humor and blood between cataract patients with and without pseudoexfoliation syndrome. C: cataract; PEX: unilateral pseudoexfoliation syndrome. (a) *p* < 0.05. (b) *p* < 0.01.

**Figure 3:**
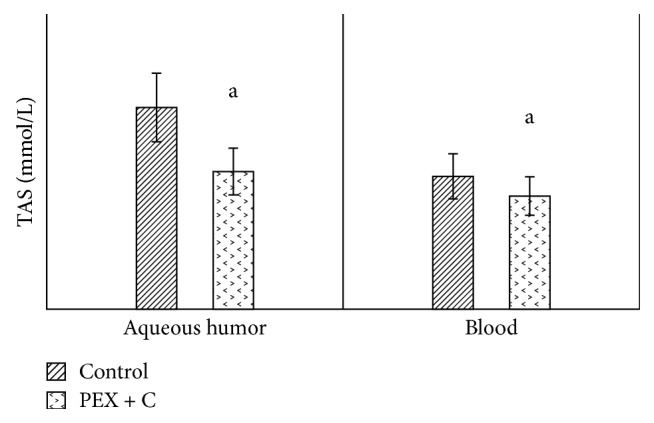
Comparison of TAS levels in aqueous humor and blood between cataract patients with and without pseudoexfoliation syndrome. C: cataract; PEX: unilateral pseudoexfoliation syndrome; TAS: total antioxidant. (a) *p* < 0.05.

**Figure 4:**
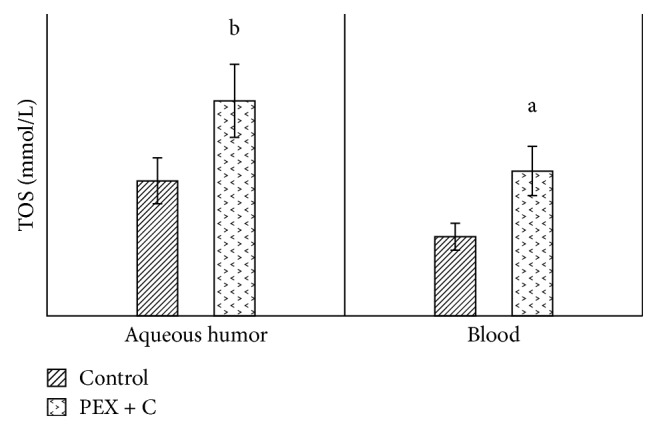
Comparison of TOS levels in aqueous humor and blood between patients with and without pseudoexfoliation syndrome. C: cataract; PEX: unilateral pseudoexfoliation syndrome; TOS: total oxidant. (a) *p* < 0.05. (b) *p* < 0.01.

**Figure 5:**
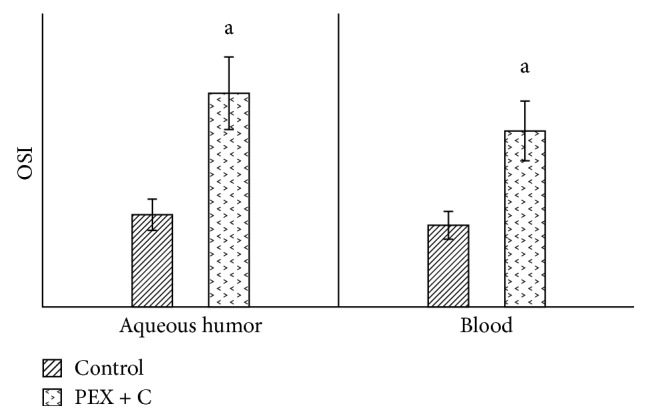
Comparison of OSI in aqueous humor and blood between patients with and without pseudoexfoliation syndrome. C: cataract; OSI: oxidative stress index; PEX: unilateral pseudoexfoliation syndrome. (a) *p* < 0.05.

The values of decorin in the aqueous humor and in the blood were significantly higher in the PEX group than in the control group (Figure 1). When we compared the aqueous humor values of decorin with the blood values, we observed statistically significantly higher levels in both the patient group and the control group (Figure 1). Similarly, when compared with the control group (patients with cataracts who did not have pseudoexfoliation), tenascin C values in both the aqueous humor and blood were significantly high in the PEX group (Figure 2). When we compared the aqueous humor values of tenascin C with the blood values, we observed that they were significantly higher in both the patient and control groups (Figure 2). Compared with the control group, the TAS values in both the aqueous humor and blood were detected to be significantly lower in the PEX group (Figure 3). When we compared the aqueous humor values of TAS with the blood values, it was observed that they were significantly higher in both the PEX and control groups (Figure 3). The values of TOS in the aqueous humor and blood were significantly higher in the PEX group than in the control group (Figure 4). When we compared the aqueous humor values of TOS with the blood values, we observed that they were significantly higher in both the PEX group and the control group (Figure 4). There was local tissue reduction of TAS activity in PEX. Compared with the control group, we found the OSI values of the PEX group to be increased in both the aqueous humor and blood, and these increases were found to be significant (Figure 5).

## 4. Discussion

Pseudoexfoliation syndrome is an eye disease in which an extracellular matrix disorder is observed. PEX is often associated with cataracts, which causes ocular involvement and systemic involvement [1]. The worldwide incidence of PEX varies according to regional population differences and genetic variations, and etiopathology maintains its place on the agenda as an important eye health problem that needs to be fully addressed [3, 32, 33]. In recent years, matrix metalloproteinases have moved to the top of the list of the most frequently studied molecules with regard to the etiopathology of this disease [7]. DEC and TN-C are glycoprotein/proteoglycan-structured matrix metalloproteinases, and a study investigating their association with PEX has not been performed yet. Oxidative stress also plays a role in the etiopathology of many diseases. Therefore, the relationship between DEC, TN-C, TAS values, TOS values, and PEX was shown for the first time in this study. However, before the possible roles of these parameters in patients with PEX are discussed individually, we must first discuss the observed demographic characteristics, since the association of both age and sex with cataracts has been revealed by many researchers in academic studies.

For example, many studies have shown that there is a significant positive correlation between age and the incidence of PEX. Moreover, the incidence after 50 years of age increases by a factor of two in each decade. Although PEX is often unilateral, it has also been shown that PEX appears in both eyes with advancing age in 13% of patients. In our study, we reported that the age averages of PEX and PEX-free cases were close to each other (no statistically significant difference) and that PEX was generally seen in only one eye.

Increased cataract prevalence is observed in patients with PEX. Both conditions occur with advanced age, and their co-occurrence is also frequent. This co-occurrence was reported with different ratios by different researchers as follows: Lumme 31%, Elibol 13.7%, Yalaz 17.7%, Drolsum 16%, Naumann 3%, Schönberr 5%, and Freyler 0.98% [34–40].

In addition, the complication rate in the PEX group was higher in this study than in the control group, but this was not statistically significant. Similarly, the complication rates in the PEX group were found to be high in many studies, but this was not statistically significant [41]. These complications, which are not statistically significant, are predicted to be due to differences in cataract morphology and the ages of the patients.

The incidence of PEX among sexes is debatable. Some studies reported that PEX can be seen more frequently in men, while other studies reported that PEX can be more frequent in women [42, 43]. However, many studies have shown that there is no significant relationship between sex and PEX [44]. There was no significant relationship between sex and PEX frequency in this present study. Therefore, TAS, TOS, TN-C, and DEC levels in this study were evaluated without sex distinction.

In this study, when levels of TAS in the aqueous humor and blood of patients with PEX were compared with those of the control group, TAS levels were significantly lower; however, TOS levels were reported to be significantly higher. We suggest that the observed decrease in TAS level and increase in TOS level in PEX syndrome increases oxidative stress, which makes cell membranes brittle, and extracellular matrix disorders may occur after increased cell destruction. This is further supported by our observed oxidative stress index values. In addition, oxidative damage is related to many pathological problems of the eye [45–47]. Several investigators have suggested that the reduction in antioxidant enzyme activities in ocular degenerative diseases may be a cause of oxidative stress in ocular degenerative diseases. Ferreira et al. conducted a study including patients with primary open-angle glaucoma. According to this study, TAS values in the aqueous humor of patients were significantly lower than those in the control group. The results of Ferreira et al. are consistent with our results, which were observed in patients with PEX syndrome [48].

A unilateral PEX group and control group (cataract patients without pseudoexfoliation) were compared in this study. DEC and TN-C values, in both aqueous humor and blood, were detected to be significantly higher in the unilateral PEX group.

DEC and TN-C are two important members of the matrix metalloproteinase class. Matrix metalloproteinases (MMPs) are involved in many physiological processes including embryogenesis and wound healing [49]. In addition, MMPs mediate cell penetration, leading proteins to pass the extracellular matrix (ECM) [50]. Presumably, MMP-2 also plays a major role in epithelial cell migration in the human eye lens. The increased levels of DEC and TN-C in PEX may be due to increased synthesis by the residual lens epithelial cells. Increased levels of these two molecules in aqueous humor in this study, when compared with blood, indicates that these two molecules are also synthesized in these tissues. In another study, TN-C expression has been reported to be upregulated in human lens epithelial cells during posterior capsule opacification (PCO) [51]. Therefore, it is considered that the levels of DEC and TN-C are increased in order to establish the tissue structure in PEX and the adaptation of individuals to PEX.

To determine whether the kits used in this study (DEC, TN-C, TAS, and TOS) can measure the values of interest in both aqueous humor and blood with the same sensitivity, the kits were tested with Aydin's previously specified assay validation experiment [52]. The kits were confirmed to measure with the same sensitivity in aqueous humor as in blood. Although kits are generally produced by manufacturers to detect biological molecules in blood, it has been reported by researchers that similar kits can be used with the same precision for many biological samples, including breast milk. The manufacturers of the kits specifically used in this study have indicated in their catalog that they can measure other biological fluids aside from blood.

As a result, PEX is a common condition in societies given the prevalence of general cataracts. When the patient and control groups were compared in this study, DEC and TN-C showed a statistically significant increase in the PEX group. In addition, the TAS level in the PEX group was statistically decreased, while the TOS level was statistically increased. In relation to the ways that these parameters may play a role in the etiopathology of PEX, we suggest that bringing these extracellular proteins, TOS levels and TAS levels back to within physiological limits can mediate the reorganization of the blood-aqueous fluid barrier and slow the progression of PEX. Additionally, these outcomes might be interrelated with a gradual deposition of fibrillary residue from the lens, ciliary body, iris, zonules, and corneal endothelium.

## Figures and Tables

**Table 1:** Demographic and cataract characteristics of patients.

Group	Age	BMI	Sex		Location		Type of cataract
			Male	Female	Right eye	Left eye	
Control (cataract)	71.64 ± 6.43	25.46 ± 2.59	4	2	2	4	Posterior subcapsular cataract
			3	2	—	5	Mature cataract
			11	13	11^a^	13^a^	Nuclear cataract
			5	4	6	3	Cortical cataract
			**23**	**21**	**19**	**25**	*Total* (*n*: 44)

PEX + cataract	70.93 ± 5.63	25.66 ± 2.46	5	2	2	5	Posterior subcapsular cataract
			2	4	2	4	Mature cataract
			12	19	17^a^	14^a^	Nuclear cataract
			—	—	—	—	Cortical cataract
			**19**	**25**	**21**	**23**	*Total* (*n*: 44)

PEX: pseudoexfoliation syndrome; BMI: body mass index. ^a^*p* < 0.05.

## Data Availability

The data used to support the findings of this study are available from the corresponding author upon request.
